# Assessment of a semi-automated protocol for multiplex analysis of sepsis-causing bacteria with spiked whole blood samples

**DOI:** 10.1002/mbo3.69

**Published:** 2013-02-18

**Authors:** Sanna Laakso, Minna Mäki

**Affiliations:** Mobidiag Ltd.Helsinki, Finland

**Keywords:** Molecular diagnostics, MRSA, sepsis, whole blood

## Abstract

Sepsis is associated with high morbidity and mortality rates worldwide. Rapid and reliable diagnostic methods are needed for efficient and evidence-based treatment of septic patients. Recently, new molecular tools have emerged to complement the conventional culture-based diagnostic methods. In this study, we used spiked whole blood samples to evaluate together two ready-to-use molecular solutions for the detection of sepsis-causing bacteria. We spiked whole blood with bacterial species relevant in sepsis and extracted bacterial DNA with the NorDiag Arrow device, using the SelectNA Blood pathogen DNA isolation kit. DNA extracts were analyzed by the polymerase chain reaction (PCR)- and microarray-based Prove-it™ Bone and Joint assay, resulting in correctly identified bacterial species with detection limits of 11–600 colony-forming unit/mL (CFU/mL). To understand the recovery losses of bacterial DNA during the sample preparation step and the capability of the PCR- and microarray-based platform to respond to the sensitivity requirements, we also determined the analytical sensitivity of the PCR and microarray platform to be 1–21 genome equivalents for the tested bacterial species. In addition, the inclusivity of the Prove-it™ Bone and Joint assay was demonstrated with methicillin-resistant *Staphylococcus aureus* (MRSA) clones carrying SCC*mec* types I, II, IV, or V and a nontypable SCC*mec* type. The proof-of-concept for accurate multiplex pathogen and antibacterial resistance marker detection from spiked whole blood samples was demonstrated by the selective bacterial DNA extraction method combined with the high-throughput PCR- and microarray-based platform. Further investigations are needed to study the promising potential of the concept for sensitive, semi-automated identification of sepsis-causing pathogens directly from whole blood.

## Introduction

Sepsis is defined as the presence of systemic inflammatory response syndrome (SIRS) in addition to a confirmed or presumed infection. It can progress to a cascade of events which increase morbidity and mortality in patients (Carrigan et al. 2004; Mancini et al. 2010). Septicemia is estimated to be the 10th leading cause of death in the United States (Minino et al. 2007) and cause approximately 20,000 deaths per day worldwide (Daniels 2011). Kumar and colleagues (2006) demonstrated a strong relationship between the delay in initiation of appropriate antimicrobial therapy and increased mortality. Rapid detection of causative microorganism(s) in blood of septic patients is thus critical for an early, evidence-based patient management.

Blood culture is the gold standard for the determination of sepsis-causing bacteria. Identification of causative pathogens and determination of antibiotic sensitivity profiles require typically 2–5 days. Recently, novel nucleic acid (NA)–based amplification methods have been developed to speed up diagnosis of sepsis. Some of these new concepts are aimed at detecting causative agents directly from whole blood without any culture periods (Klouche and Schröder 2008; La Scola and Raoult 2009; Mancini et al. 2010; Paolucci et al. 2010). Identification of bacteria from whole blood eliminates time-consuming culture steps, but sets high requirements for the sample preparation method in terms of efficient recovery and purification of bacterial DNA and for the NA amplification methods in terms of sensitive detection of bacterial DNA. The amount of infecting bacteria in clinically significant bacteremia is low (Ecker et al. 2010) whereas levels of potentially interfering substances, such as human DNA, heme, and anticoagulants in the blood collection tube, e.g. Ethylenediaminetetraacetic acid (EDTA), are high (Al-Sould et al. 2000).

The aim of this study was to bring together two ready-to-use solutions for faster sepsis diagnostics; a semi-automated sample preparation method for whole blood samples used in conjunction with the high-throughput polymerase chain reaction (PCR)- and microarray-based bacterial identification method. We used the SelectNA Blood pathogen DNA isolation kit (Molzym, Germany) together with the automated NorDiag Arrow (NorDiag, Norway) extraction device for isolation of bacterial DNA. The Prove-it™ Bone and Joint (Mobidiag, Finland), a PCR- and microarray-based assay, was used for the analysis of DNA extracts.

SelectNA Blood pathogen DNA isolation kit used with the NorDiag Arrow extraction device provides a semi-automated solution for isolation and concentration of bacterial and fungal DNA from whole blood samples. The principle of this method is first to selectively lyse human cells with chaotropic buffers, after which released human DNA is digested. Bacterial and fungal cells remain intact during these steps. They are concentrated by centrifugation and lysed by muralytic enzymes before DNA extraction (Disque et al. 2004; Horz et al. 2010) with the NorDiag Arrow extraction device, which utilizes magnetic particle–based extraction technology (NorDiag: Application Note An-19-10). The described sample preparation method and device are the same as in the SepsiTest™ SelectNA assay (Molzym, Germany).

The Prove-it™ Bone and Joint assay belongs to the Prove-it™ test family which consists of a broad-range bacterial PCR- and microarray-based platform. In the Prove-it™ Bone and Joint assay especially the PCR protocol has been developed further from the Prove-it™ Sepsis assay to be more suitable for clinical sample matrices (e.g., osteoarticular fluids or bone specimens) which contain low amounts of bacteria and high levels of interfering substances (L. Metso, M. Mäki, P. Tissari, V. Remes, P. Piiparinen, J. Kirveskari, E. Tarkka, V.-J. Anttila, M. Vaara, and K. Huotari, unpubl. ms.). The platform's microarray has broad pathogen coverage; it targets over 60 clinically relevant bacterial species in a single reaction and it has been evaluated using over 3300 positive blood cultures to be 99% specific in identification of bacterial species in the clinical setting. The platform allows simultaneous identification of staphylococcal species and the methicillin-resistance gene *mecA* (Järvinen et al. 2009; Tissari et al. 2010; Laakso et al. 2011).

Antibacterial resistance can have a major influence on treatment outcome of septic patients. Turnidge (2003) concluded that the presence of bacterial resistance approximately doubles the mortality rate associated with sepsis. Methicillin-resistant *Staphylococcus aureus* (MRSA) has become one of the most commonly identified antibiotic-resistant bacteria in many parts of the world. Methicillin resistance in *Staphylococcus* species arises principally by the acquisition of a highly mobile element, the staphylococcal cassette chromosome, SCC*mec*. The SCC*mec* element carries the *mecA* gene, which encodes penicillin-binding protein PBP2a, the main causal factor of methicillin resistance. Currently, 11 main types of SCC*mec* elements (types I–XI) and several variants have been identified based on differences in structure and size (Peng et al. 2010; Shore et al. 2011). This genetic variation poses challenges to the correct detection of MRSA using NA-based methods. Hence, we also studied the Prove-it™ Bone and Joint assay's inclusivity for MRSA clones with different SCC*mec* variants.

## Materials and Methods

### Samples

Six well characterized clinical isolates and two reference strains from the American Type Culture Collection (ATCC, VA) were used for the experiments. *Escherichia coli* (ATCC 25922 strain and one clinical isolate), *Klebsiella pneumoniae* (a clinical isolate), *S. aureus* (ATCC 25923 strain), MRSA (a clinical isolate), *Streptococcus agalactiae* (a clinical isolate), *Enterococcus faecalis* (a clinical isolate), and *Listeria monocytogenes* (a clinical isolate) were cultivated overnight on blood agar plates at 37°C under aerobic conditions after which the cells were used for further analysis. In addition, 18 well characterized MRSA clones collected from Finland (Table 1) were cultured under the described conditions.

**Table 1:** Inclusivity of the Prove-it™ Bone and Joint assay for the MRSA clones

SCC*mec* type	MRSA clones using FIN codes^1^	Number of tested isolates	Analysis results by Prove-it™ Bone and Joint assay
I	FIN-21	4	*Staphylococcus aureus*, *mecA*
II	FIN-3	1	*S. aureus*, *mecA*
	FIN-16b	1	*S. aureus*, *mecA*
IV	FIN-7	1	*S. aureus*, *mecA*
	FIN-12	1	*S. aureus*, *mecA*
	FIN-11	1	*S. aureus*, *mecA*
	FIN-37	1	*S. aureus*, *mecA*
	FIN-4	2	*S. aureus*, *mecA*
IV or V	FIN-10	3	*S. aureus*, *mecA*
V	FIN-22	1	*S. aureus*, *mecA*
	FIN-36	1	*S. aureus*, *mecA*
Nontypable SCC*mec*^2^	FIN-3b	1	*S. aureus*, *mecA*
Total		18	

MRSA, methicillin-resistant *Staphylococcus aureus*; SCC*mec*, staphylococcal cassette chromosome mec.

1 Finnish nomenclature for MRSA clones categorized on the basis of pulsed field gel electrophoresis (PFGE) clusters (Vainio, A. 2012. Molecular methods for the epidemiological analysis of methicillin-resistant *Staphylococcus aureus*. Ph.D. thesis).

2 SCC*mec* carries genes *ccrA1*, *ccrA2*, and the class B type of *mec*.

### Sample preparation and DNA extraction

#### Preparation of the samples for the measurements of Prove-it™ Bone and Joint assay's analytical sensitivity

After bacterial culturing, a few colonies of *E. coli*, *K. pneumoniae*, *S. aureus*, MRSA, *S. agalactiae*, *E. faecalis*, and *L. monocytogenes* were picked from the blood agar plates for DNA extraction with the NucliSENS®easyMAG® (bioMérieux, France) device using the Generic 2.0.1 protocol according to the manufacturer's instructions. DNA concentrations of the extracted samples were determined by using a NanoDrop spectrophotometer (Thermo Fisher Scientific, Waltham, MA) according to the manufacturer's instructions. Dilution series from bacterial DNA were prepared by 10-fold dilutions down to 33 fg/μL. Using the Prove-it™ Bone and Joint assay, 1.5 μL from the concentrations of 3.3 pg/μL, 330 and 33 fg/μL (corresponding to approximately 10^3^, 10^2^, and 10^1^ genome equivalents [GE]) from each bacterium were tested as duplicates. DNA concentrations of *E. coli* and *S. aureus* were determined using a real-time PCR method. DNA was diluted to the final concentrations of 10^3^, 10^2^, 10^1^, and 1 GEs and samples were tested as duplicates.

#### Preparation of MRSA samples for the inclusivity experiments

After culturing of MRSA clones, a few bacterial colonies were picked from the blood agar plate and suspended in 100 μL of 1× phosphate buffered saline (PBS). After centrifugation (at 3000 rpm for 3 min), the supernatant was discarded and the pellet was suspended in 100 μL of Tris-EDTA (TE) buffer. Two heating steps of 95°C for 5 min were performed sequentially with a 2 minutes cooling step between them. Finally, the solution was centrifuged (at 13,000 rpm for 10 min) and the supernatant containing DNA was collected. The DNA concentration was measured using a NanoDrop spectrophotometer (Thermo Fisher Scientific). DNA concentrations were adjusted to 333 pg/μL or to 3.33 pg/μL and 1.5 μL of the DNA dilutions (corresponding to approximately 10^5^ and 10^3^ GEs) and were used for analysis with the Prove-it™ Bone and Joint assay.

#### Preparation and extraction of spiked whole blood samples

**Figure 1:**
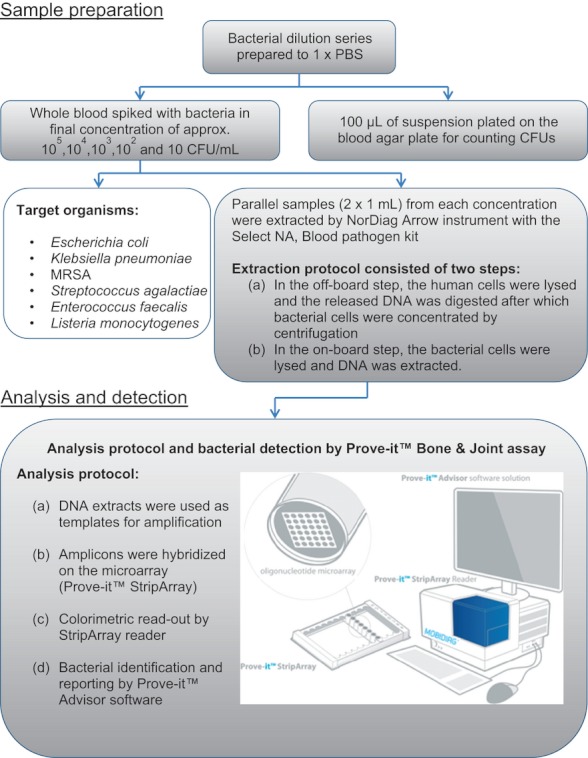
Flow diagram of the workflow of the tested concept.

One milliliter of whole blood from healthy volunteers drawn into Vacutainer® blood collection tubes (Becton Dickinson, Franklin Lakes, NJ) and stored frozen was spiked with *E. coli*, *K. pneumoniae*, MRSA, *S. agalactiae*, *E. faecalis*, or *L. monocytogenes*. Bacterial colonies were suspended into 1× PBS and the optical density (OD) of the bacterial suspension was measured at 625 nm by a spectrophotometer (BioPhotometer, Eppendorf AG, Hamburg, Germany). The density of the bacterial suspension was adjusted to an absorbance (A) of 0.132 which corresponds to McFarland 0.5 and is approximately 1.5 × 10^8^ cells/mL (McFarland Turbidity Standard No. 0.5, Becton Dickinson). The series of 10-fold dilutions were prepared from bacterial suspensions and 100 μL from each dilution was plated onto a blood agar plate. The plates were incubated at 37°C overnight for counting colony-forming units (CFU). The final bacterial concentrations in spiked whole blood samples were approximately 10^5^, 10^4^, 10^3^, 10^2^, and 10 CFU/mL. Duplicate samples were prepared from each concentration. One milliliter of each prepared sample was used for DNA extraction with the NorDiag Arrow (NorDiag, Norway) extraction device in conjunction with the SelectNA Blood pathogen DNA isolation kit (Molzym, Germany) according to the manufacturer's instructions. The protocol consisted of a short manual step where the human cells were lysed and human DNA was digested, followed by automated DNA extraction with the NorDiag Arrow device. The workflow for sample preparation and bacterial identification is described in Figure 1.

### Analysis

#### Prove-it™ Bone and Joint assay

DNA from extracted samples was analyzed by the Prove-it™ Bone and Joint StripArray assay research-use-only version (Mobidiag, Finland). Briefly, bacterial detection is based on broad-range PCR and microarray technology with colorimetric detection. Proprietary primers were used for amplification of specific regions of the bacterial topoisomerase genes *gyrB* and *parE*, and the methicillin-resistance gene *mecA* (Järvinen et al. 2009). For the amplification step which was carried out in a Mastercycler® *epgradient S* thermal cycler (Eppendorf, Germany), 1.5 μL of the DNA extract and 13.5 μL of PCR mixture were used. After the amplification step, 5 μL of amplicons were transferred onto the Prove-it™ StripArray microarray for hybridization. Positive hybridization–based reactions were detected and reported by the StripArray Reader and the Prove-it™ Advisor analysis software (version 1.1.0.0). The microarrays were automatically analyzed and the target identification result was generated using specific built-in rules and parameters in the Prove-it™ Advisor software. The result consisted of the name(s) of identified bacterial target(s) and detailed information about data parameters, such as signal intensities and the number of identified oligonucleotide probes for each target. The analytical sensitivity and inclusivity analysis of the Prove-it™ Bone and Joint assay were performed by the described protocol. Whole blood samples were analyzed with a slightly modified Prove-it™ Bone and Joint protocol where the volume of PCR template and the volume of amplicons for the hybridization step were optimized to be 5 and 10 μL, respectively.

## Results

### Sensitivity of the Prove-it™ Bone and Joint assay

In the first phase, we studied the capacity of Prove-it™ Bone and Joint assay to meet the sensitivity requirements for whole blood sepsis diagnostics. The analytical sensitivity of the Prove-it™ Bone and Joint assay was studied using bacterial DNA extracts of characterized clinical strains. DNA concentrations representing approximtely 10^3^, 10^2^, and 10^1^ GEs for *K. pneumoniae*, MRSA, *S. agalactiae*, *E. faecalis*, and *L. monocytogenes* and approximately 10^3^, 10^2^, 10^1^, and 1 GEs for *E. coli* and *S. aureus* were analyzed as duplicates using the Prove-it™ Bone and Joint assay. The limit-of-detection (LOD) was determined to be the lowest amount of GE which, when added to the PCR, led to a bacterial identification on the microarray for at least one of the tested duplicates. The result interpretation and reporting was conducted automatically by the Prove-it™ Advisor microarray analysis software.

All bacteria were correctly identified and the exact LODs were calculated to be 1 GE for *E. coli*, 8 GE for *K. pneumoniae*, 11 GE for *S. aureus*, 15 GE for *E. faecalis*, 16 GE for *L. monocytogenes*, 17 GE for MRSA, and 21 GE for *S. agalactiae* (Table 2). Calculations were based on the size of the respective bacterial genome (URI Genomics and Sequencing Center 2004). *Escherichia coli*, *K. pneumoniae*, MRSA, *S. agalactiae*, and *L. monocytogenes* samples were successfully identified and reported from both duplicates from all tested GE concentrations. Also, *S. aureus* and *E. faecalis* were identified from both duplicates in the higher GE concentrations (approximately 10^3^ and 10^2^ GEs), but the bacterial findings were reported only from one duplicate from the LOD concentration (approximately 10^1^ GE). However, when the microarray image was studied in detail, four of the eight *S. aureus* specific capture oligos on the microarray were detected from the other duplicate of the LOD concentration, indicating the amplification and detection of *S. aureus* that did not exceed the built-in identification thresholds of the Prove-it™ Advisor microarray analysis software.

**Table 2:** Analytical sensitivity of the Prove-it™ Bone and Joint assay defined by the lowest amount of GE added to the PCR reaction, which led to the correct bacterial identification

Bacterial species	Detection limit (GE)
*Escherichia coli*	1
*Klebsiella pneumoniae*	8
*Staphylococcus aureus*	11
Methicillin-resistant *Staphylococcus aureus*	17
*Streptococcus agalactiae*	21
*Enterococcus faecalis*	15
*Listeria monocytogenes*	16

GE, genome equivalents; PCR, polymerase chain reaction.

### Analysis of spiked whole blood samples

In the second phase, we used spiked EDTA-blood samples to investigate the performance of the selected sample preparation method together with the PCR and microarray analysis. One milliliter of five spiked whole blood samples per each bacterium (*E. coli*, *K. pneumoniae*, MRSA, *S. agalactiae*, *E. faecalis*, and *L. monocytogenes*) in the final concentrations of approximately 10^5^, 10^4^, 10^3^, 10^2^, and 10^1^ CFU/mL were used for the analysis. Duplicates were tested at each concentration. DNA extraction was conducted with the SelectNA Blood pathogen DNA isolation kit and the NorDiag Arrow extraction device, after which the DNA extracts were analyzed by the Prove-it™ Bone and Joint assay. The LODs were determined to be the lowest amount of CFUs spiked into the blood sample which led to a correct bacterial identification reported by the Prove-it™ Advisor microarray analysis software from one or both duplicates.

All bacteria were correctly identified and the determined LODs were 11 CFU/mL for *E. coli*, 13 CFU/mL for *E. faecalis*, 68 CFU/mL for *K. pneumoniae*, 86 CFU/mL for MRSA, 250 CFU/mL for *L. monocytogenes*, and 600 CFU/mL for *S. agalactiae* (Table 3). Comparing bacterial detection from duplicates, MRSA was successfully identified from both duplicates in the LOD concentration. *Escherichia coli*, *K. pneumoniae*, *S. agalactiae*, *E. faecalis*, and *L. monocytogenes* were successfully detected from one duplicate while the other duplicate remained negative with the exception of *K. pneumoniae*. When the microarray image was studied in detail, four of the eight *K. pneumoniae* specific capture oligos were detected on the microarray indicating the amplification of *K. pneumoniae*. The total assay time for the concept was around 6 h, including hands-on time.

**Table 3:** Combined performance of the SelectNA Blood pathogen DNA isolation kit used together with the NorDiag Arrow extraction device and the Prove-it™ Bone and Joint assay

Bacterial species	Detection limit^1^ (CFU/mL)
*Escherichia coli*	11
*Klebsiella pneumoniae*	68
Methicillin-resistant *Staphylococcus aureus*	86
*Streptococcus agalactiae*	600
*Enterococcus faecalis*	13
*Listeria monocytogenes*	250

CFU, colony-forming unit; LOD, limit-of-detection.

1 LOD is defined to be the lowest amount of bacteria spiked into whole blood (CFU/mL), which led to correct bacterial identification.

### Inclusivity of the Prove-it™ Bone and Joint assay for MRSA clones carrying different *SCCmec* types

In the Prove-it™ Bone and Joint assay, the MRSA identification is based on the detection of the *S. aureus*-specific topoisomerase (*gyrB)* gene region and the methicillin-resistance gene *mecA* (Järvinen et al. 2009). We studied the inclusivity of Prove-it™ Bone and Joint assay for epidemic MRSA clones using 18 different MRSA isolates collected from Finland. Seventeen MRSA clones carried the SCC*mec* types I, II, IV, or V and one clone possessed a nontypable SCC*mec* complex. Concentrations of approximately 10^5^ and 10^3^ GEs for all MRSA clones were used for the Prove-it™ Bone and Joint analysis. From all the tested MRSA clones, both *S. aureus* and the *mecA* gene findings were reported (Table 1).

## Discussion

New molecular diagnostic solutions complementing time-consuming blood culture methods can facilitate identification of sepsis-causing microbes directly from blood and thus direct patient management toward earlier, pathogen-driven treatment options. Afshari and coworkers (2012) summarized the current, commercially available molecular techniques for sepsis diagnostics with their advantages and disadvantages. From the available ready-to-use solutions, we selected the cost-effective, high-throughput Prove-it™ platform and the semi-automated sample preparation method, of which the same principle has also been used in the SepsiTest™ SelectNA (Molzym, Germany) assay. We assessed the performance of the combined concept for identification of sepsis-causing bacteria using the spiked whole blood samples. The sample preparation consisted of the SelectNA Blood pathogen DNA isolation kit which was used together with the NorDiag Arrow extraction device. For the analysis with Prove-it™ Bone and Joint assay, we used a protocol optimized for the highly sensitive pathogen detection from various clinical sample matrices containing a low amount of bacteria. The LODs of 11–600 CFU/mL of spiked EDTA-blood were determined for *E. coli*, *K. pneumoniae*, MRSA, *S. agalactiae*, *E. faecalis*, and *L. monocytogenes*, demonstrating the proof-of-concept for the sensitive and accurate performance of the combined solutions.

When adapting a PCR-based protocol in routine sepsis diagnostics, a prerequisite for sensitive analysis is an efficient sample preparation step, including DNA extraction. In addition, automated and less labor-intensive solutions, which may reduce human errors and possible contaminations while improving precision, reproducibility, and traceability, are also preferred (Regueiro et al. 2010; Struelens 2010). Whole blood is known to be a very complex sample matrix for PCR applications. In sepsis cases, the amount of infecting bacteria in blood is very low (Ecker et al. 2010) and levels of interfering substances are high (Al-Sould et al. 2000). In recent years, a number of sample preparation solutions have become available, but only few of them have been evaluated successfully for sensitive microbial DNA extraction from whole blood (Regueiro et al. 2010; Wiesinger-Mayr et al. 2011). The Molzym solution for selective bacterial isolation using manual kits has been proven to perform well with whole blood samples obtained from sepsis patients, albeit using other downstream detection methods for microbial detection than the one used in this study (Wellinghausen et al. 2009; Wiesinger-Mayr et al. 2011). The SelectNA Blood pathogen DNA isolation kit, used together with the NorDiag Arrow extraction device, provides a semi-automated solution for sample preparation, utilizing the same procedure as is used in the SepsiTest™ SelectNA assay (Molzym, Germany). The NorDiag Arrow extraction device has been considered to be easy to implement in clinical laboratories, allowing 1–12 simultaneous preparations of samples with reduced hands-on time (Laakso et al. 2011).

**Figure 2:**
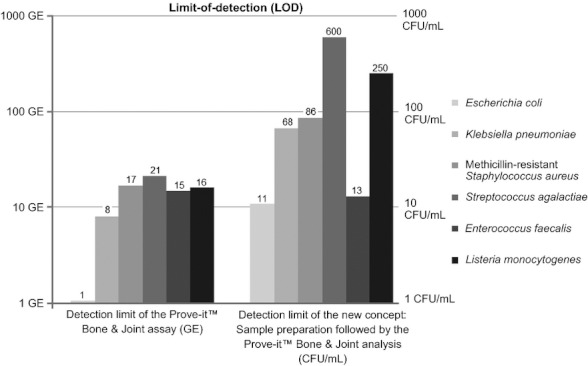
Comparison of the detection limits. Analytical sensitivity of Prove-it™ Bone and Joint assay compared with the detection limits (colony-forming units per mL [CFU/mL]) of the tested concept which includes the sample preparation steps for whole blood in addition to the analysis by the Prove-it™ Bone and Joint assay. Values represent the lowest concentration (genome equivalents [GE] or CFU/mL) where bacteria from one or both duplicates were identified.

We selected both gram-negative and gram-positive bacterial species relevant in sepsis for the evaluation of the combined assays. The LODs measured for the tested concept varied from 11 to 600 CFU/mL of spiked EDTA-blood, depending on the bacterial species. When these LODs were compared with the analytical sensitivity of the Prove-it™ Bone and Joint assay, they were in a similar range (11–86 CFU/mL vs. 1–16 GE) with the exception of *L. monocytogenes* (250 CFU/mL vs. 16 GE) and *S. agalactiae* (600 CFU/mL vs. 21 GE) (Fig. 2). We acknowledge that CFU/mL and GE are not fully comparable units and that the comparison between these units is not unambiguous, but the data indicate possible inefficient removal of PCR inhibitors originating from blood and/or recovery losses of bacterial DNA during the sample preparation step. Overall the obtained LODs were similar to those of SepsiTest™ (Gebert et al. 2008; Mühl et al. 2010) and LightCycler® SeptiFast Test MGRADE (F. Hoffmann-La Roche, Germany), the most commonly used and evaluated solutions for whole blood sepsis diagnostics. Lehmann and coworkers (2008) have determined that the analytical sensitivity of LightCycler® SeptiFast Test MGRADE varies from 3 to 100 CFU/mL, depending on the bacterial species.

The number of recoverable CFU of bacteria in blood is typically around 1–30 CFU/mL in adult septic patients while in children it can exceed 100 CFU/mL (Ecker et al. 2010). In order to meet these sensitivity requirements for all the bacterial species the concept could identify (>60 targets), the analytical sensitivity of the concept could be improved by increasing the sample volume and/or decreasing the elution volume of DNA extracts during the sample preparation. Furthermore, the volume of template in the PCR- and microarray-based assay can also be increased if the removal of putative PCR inhibitors is efficient and their presence in the eluate can be ruled out. As sample volume we used 1 mL of blood according to the manufacturer's instructions, but Mühl and colleagues (2011) have demonstrated that using a larger blood volume in the SelectNA Blood pathogen–based sample preparation step increases the sensitivity of PCR-based downstream analysis. On the basis of their data, 79% (11/14) of blood samples taken from patients suffering from SIRS, sepsis, or neutropenic fever were positive in PCR-based analysis using 5 mL of blood in contrast to only a 50% (7/14) positivity rate using 1 mL of blood. As elution volume, we used 100 μL according to the manufacturer's instruction, from which a relatively low amount (1–5 μL) was used as template for the PCR- and microarray-based reaction.

Antimicrobial-resistant bacteria have emerged worldwide and it has been shown that antimicrobial resistance can have a major influence on treatment outcome of septic patients (Turnidge 2003). Patient management faces challenges with rising rates of MRSA, especially in healthcare-associated and community-acquired MRSA infections, with potentially serious consequences for patients and extra burden on healthcare resources (Huttunen and Aittoniemi 2011). We investigated the inclusivity of the Prove-it™ Bone and Joint assay for epidemic MRSA clones carrying different SCC*mec* complexes. Tissari and colleagues (2010) have previously shown that the clinical sensitivity and specificity of the Prove-it™ Sepsis assay were 100% for MRSA bacteremia. Now using the almost identical microarray, we demonstrated that 17 clinically important clones carrying SCC*mec* I, II, IV, or V and one nontypable SCC*mec* were accurately identified by the Prove-it™ Bone and Joint assay with regard to the *S. aureus gyrB* and the *mecA* gene fragments.

In conclusion, we reported here the proof-of-concept for identification of sepsis-causing bacteria directly from spiked whole blood using two ready-to-use solutions already available for diagnostic use. It appears that the combination of an automated bacterial DNA extraction method (NorDiag Arrow with the SelectNA Blood pathogen DNA isolation kit) and the Prove-it™ Bone and Joint assay could offer a valuable tool for sensitive, multiplex analysis of bacteria and their antibacterial resistance markers in a timely manner. This in turn could speed up some steps in the diagnostic strategy of septic patients. Thus, the concept deserves further optimization and intensive testing. Furthermore, the applicability of the concept for diagnosis of sepsis with real patient samples in clinical settings remains to be elucidated.

## Conflict of Interest

SL and MM were affiliated with Mobidiag, the manufacturer of Prove-it™ Bone and Joint assay used in this study.
